# Current skin care practices in very low birth weight infants in German Neonatal Intensive Care Units – results from a cross-sectional survey

**DOI:** 10.1186/s40348-026-00237-0

**Published:** 2026-04-29

**Authors:** Janina Marissen, Regina Thoma, Christoph Härtel

**Affiliations:** 1 Department of Pediatrics, University Hospital Würzburg, Würzburg, Germany; 2 German Center for Infection Research (DZIF), Partner Site Hamburg-Lübeck- Borstel-Riems, Lübeck, Germany; 3 Nursing Directorate, University Hospital Würzburg, Würzburg, Germany

**Keywords:** Preterm infant, Neonate, Skin care, VLBWI, NICU, Survey

## Abstract

**Background:**

The skin is the largest epithelial organ and a critical barrier. However, in preterm infants, particularly very low birth weight infants (VLBWI, < 1500 g), it is immature and highly susceptible to injury and infection. Despite growing awareness of neonatal skin care, evidence-based guidelines for this vulnerable population are lacking. This study aimed to provide an overview of current skin care practices in German Neonatal Intensive Care units (NICUs) and compare them with other European centers.

**Methods:**

A cross-sectional, web-based survey was distributed to German level III NICUs (*n* = 70) and selected European centers (*n* = 10) in May 2025. The questionnaire comprised 20 items covering skin risk assessment and skin care routines, bathing, skin antisepsis, and perceived challenges. Responses were analyzed descriptively.

**Results:**

The overall response rate was 73.8% (*n* = 59). Responses were analyzed from 48 German NICUs and eight European centers. Among German centers, only 14.6% (*n* = 7) reported applying skin risk assessment tools, and 16.7% (*n* = 8) reported standardized skin care protocols. A dexpanthenol-containing cream was the most commonly used skin care product. Use of other products varied greatly, while 39.6% of German centers (*n* = 19) reported using no skin care products at all. Bathing was practiced in 77.1% of NICUs (*n* = 37), with frequency and use of additives varying. Octenidine 0.1% and its combinations were the main products for skin antisepsis. Major challenges perceived by neonatologists included high skin vulnerability (87.5%), issues with adhesives (72.9%), risk of infection (64.5%), and limited or unclear evidence in skin care of VLBWI (54.2%). Criteria for product selection included clinical evidence (60.4%), practical experience (60.4%), and hygiene regulations (54.2%). Comparison with reports from the other European countries revealed both notable similarities and considerable variations in practices across the international context.

**Conclusion:**

Skin care practices for VLBWI in German and European NICUs vary widely, with limited use of standardized protocols or risk assessment tools. High skin vulnerability, issues with adhesives and infection risk remain the main challenges. These findings underscore the urgent need for evidence-based guidelines to optimize skin care in this high-risk population.

**Supplementary Information:**

The online version contains supplementary material available at 10.1186/s40348-026-00237-0.

## Background

The skin is the largest epithelial organ and plays a central role in numerous processes, serving as a critical interface between the individual and the external environment. In preterm infants, particularly in very low birth weight infants (VLBWI; birth weight < 1500 g), skin structure and function are markedly immature [1]. Depending on gestational age, the epidermis is significantly thinner, with fewer layers of stratum corneum, reduced desmosomes and fewer underlying structural fibers in the dermis [2]. Protective components such as vernix caseosa begin to develop only after approximately 28 weeks of gestation. Consequently, skin biomarkers in preterm infants likely differ substantially from those in healthy term neonates [2, 3].

Although the transition from the intrauterine aqueous environment to the comparatively drier incubator air promotes skin maturation [4], VLBWI remain at high risk for skin injury and infection [5, 6]. Contributing factors include the use of adhesives for respiratory support, continuous monitoring devices, and invasive procedures such as the placement of central intravenous lines [2, 5].

A large survey-based study of more than 800 Neonatal Intensive Care Units (NICUs) worldwide reported that medical adhesive-related skin injuries were among the most frequently observed skin injuries in extremely preterm infants < 28 weeks (38%) [5]. In this survey, adherence to local skincare guidelines and performing skin assessments at least every four hours were associated with lower rates of skin injury. In addition, the use of oil-based-ointments was associated with reduced odds for certain types of skin injuries [5]. Regarding infection, a Cochrane meta-analysis of 22 trials found little or no clear effects of topical ointments and creams on invasive infection or mortality, although there was some evidence that oil-based emollients may reduce invasive infection in low- and middle-income settings [7]. Data from high-income countries are scarce. Emollient use in these settings is generally lower, and practices vary [5]. However, a large multicenter study investigating the effects of coconut oil on late-onset sepsis in extremely preterm infants is currently underway in Australia and New Zealand (COSI-2 trial) [8].

Despite growing awareness of the importance of skin development and targeted skin care strategies in preterm infants, many aspects of optimal supportive skin care remain unclear, and evidence-based guidelines for this vulnerable cohort are still lacking [9, 10]. A German expert standard for skin care, for example, excludes preterm infants in their recommendations [10]. In other regions, such as Switzerland and Western Australia, skin care guidelines have been implemented, some of which aim to harmonize skin care practices across selected national centers [11–13]. As no standardized skin care protocols or evidence-based guidelines for VLBWI exist in Germany, the aim of the survey was to obtain an overview of current skin care practices in German NICUs. Based on these updated data, the current status of practical implementation as well as the need for further evidence-based research projects can be assessed.

## Methods

We conducted a cross-sectional survey using a web-based questionnaire (evasys GmbH). The questionnaire was developed by the study team and internally assessed for clarity and relevance prior to distribution. The final version comprised 20 items including single-choice, multiple-choice, and free-text responses. The full questionnaire is provided in the Supplementary Material.

The questionnaire was distributed electronically via email in May 2025 to medical directors of Level III NICUs in Germany (German classification level I; *n* = 70) and through the I4 section of the European Society for Paediatric Research (ESPR; *n* = 10). Replies were collected over a five-week episode between May and June 2025. One response per center was requested.

Participation was voluntary and anonymous. No identifiable personal or institutional data were collected. Clinicians were asked to report their country and the annual number of VLBWI treated in their center per year. All submissions were screened for duplicate entries; no duplicates were found. Three responses, that lacked information on country of origin were excluded from the analysis.

Due to the anonymous and voluntary nature of the clinician survey, which did not involve any patient or any identifiable data, no formal ethical approval was required following consultation with the local ethics committee.

Data were analyzed descriptively using IBM SPSS Statistics (version 31). Binary and nominal variables are presented as n and %. To estimate the annual number of VLBWIs covered by participating centers, midpoint values were derived from categorical ranges of treated VLBWI. Minimum and maximum estimation was added. We estimated approximately 9,000–10,000 VLBWI born annually in Germany [14]. For exploratory subgroup analysis, centers were categorized into those treating < 50 VLBWI/year (*n* = 26, “smaller centers”), and those treating > 50 VLBWI/year (*n* = 22, “larger centers”), restricted to German centers. Group comparisons were performed using Fisher´s exact test; however, no statistically significant differences were observed (all *p* > 0.05). Thus, only trends are described in the result section.

Figures were created using GraphPad Prism (version 10.6.1).

## Results

We received a total of 59 responses, corresponding to an overall response rate of 73.8%. Most reports originated from Germany (*n* = 48; 81.4%), with additional responses from other European countries (*n* = 8; 13.6%); including Italy (*n* = 3), Poland (*n* = 2), Denmark (*n* = 1), Spain (*n* = 1), and Belgium (*n* = 1). Three submissions (5%) did not include information on the country of origin and were therefore excluded from further analysis.

German and other European country data were analyzed separately. Table 1 presents the number of VBWI treated annually by the participating German centers. Most German centers (*n* = 34; 70.9%) reported caring for between 25 and 75 VLBWI per year.

**Table 1:** Very Low Birth Weight Infants (VLBWI) treated annually in the German centers responding to the survey

VLBWI treated annually	Number of centers (%)
< 25	6 (12.5)
25–50	20 (41.7)
51–75	14 (29.2)
76–100	4 (8.3)
> 100	4 (8.3)

In contrast, the majority of other European centers (*n*=5; 62.5%) reported treating over 75 VLBWI annually in their NICUs

### Skin risk assessment scores and standardized skin care protocols

Only seven centers (14.6%) reported using skin risk assessment tools in their units. Among these, the Neonatal Skin Risk Assessment Score (*n* = 2), Swiss Neonatal Skin Score, Braden Q Scale, a local score, and photo documentation (*n* = 1 each) were mentioned. Of the centers that used a risk assessment tool, four (57.1%) applied it daily, one (14.3%) several times a week, and two (28.6%) on an as-needed basis.

Regarding standardized skin care protocols, only eight centers (16,7%) reported having a dedicated skin care protocol for preterm infants, whereas the remaining 40 centers (83.3%) did not.

When comparing smaller and larger centers, smaller centers more often reported having a skin care protocol (23% vs. 9%). The use of skin risk assessment tools (*n* = 2; 25%) and standardized skin care protocols (*n* = 2; 25%) was slightly more common in other European countries, but overall remained low.

### Skin care products

**Fig. 1:**
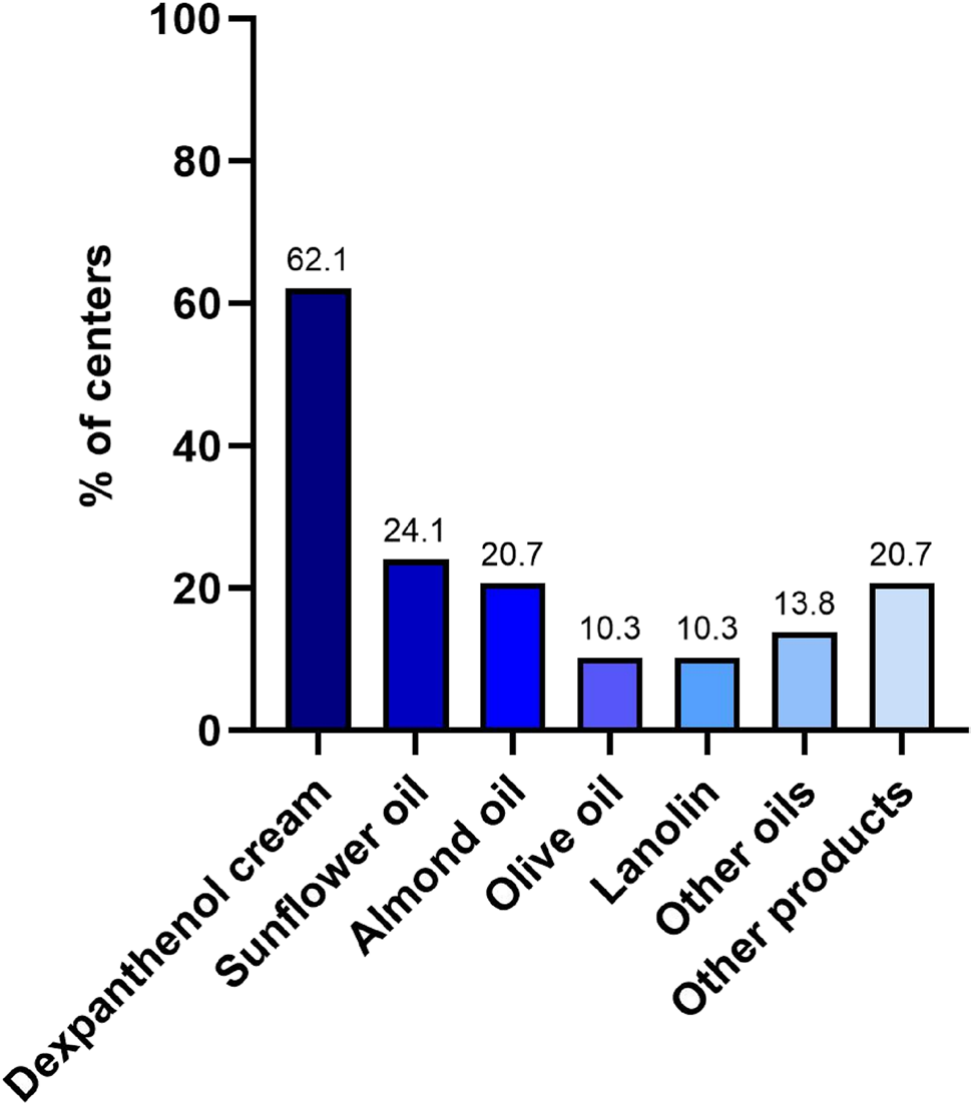
Use of skin care products in Germany

When asked about skin care products, 19 centers (39.6%) reported not using any. Among the 29 centers (60.4%) that did use skin care products, Bepanthen^®^, a dexpanthenol-containing cream, was the most commonly reported, used at 62.1% (*n* = 18) of these units. The distribution of skin care products among centers that apply them is shown in Fig. 1.

Reports on the use of skin care products among 29 centers that applied any products, with multiple responses allowed. Dexpanthenol cream refers to Bepanthen^®^. Other products reported included Avène^®^ cream, individual cream, caleo^®^ cream, zinc lanolin, Physiogel^®^, DAC^®^ cream, and other oils such as corn germ oil, Hipp^®^ baby oil, Töpfer^®^ oil, and jojoba oil (*n* = 1 response per product).

When comparing smaller and larger centers, smaller centers less often reported using no routine products (35% vs. 45%). Sunflower oil tended to be used more often in smaller centers (35% vs. 8%, if any skin care product was used). Most other European centers (*n* = 6; 75%) reported not using any routine skin care products, although some reported using Bepanthen^®^, lanolin, or vitamin E.

### Bathing practices

Among the German centers, 37 (77.1%) reported that bathing was routinely practiced in their NICUs. The frequency of bathing varied: weekly in 35.1%, twice a week in 16.2%, once to twice a week in 5.4% and without a set schedule in 21.6% of centers. Eight centers did not report any frequency.

**Fig. 2:**
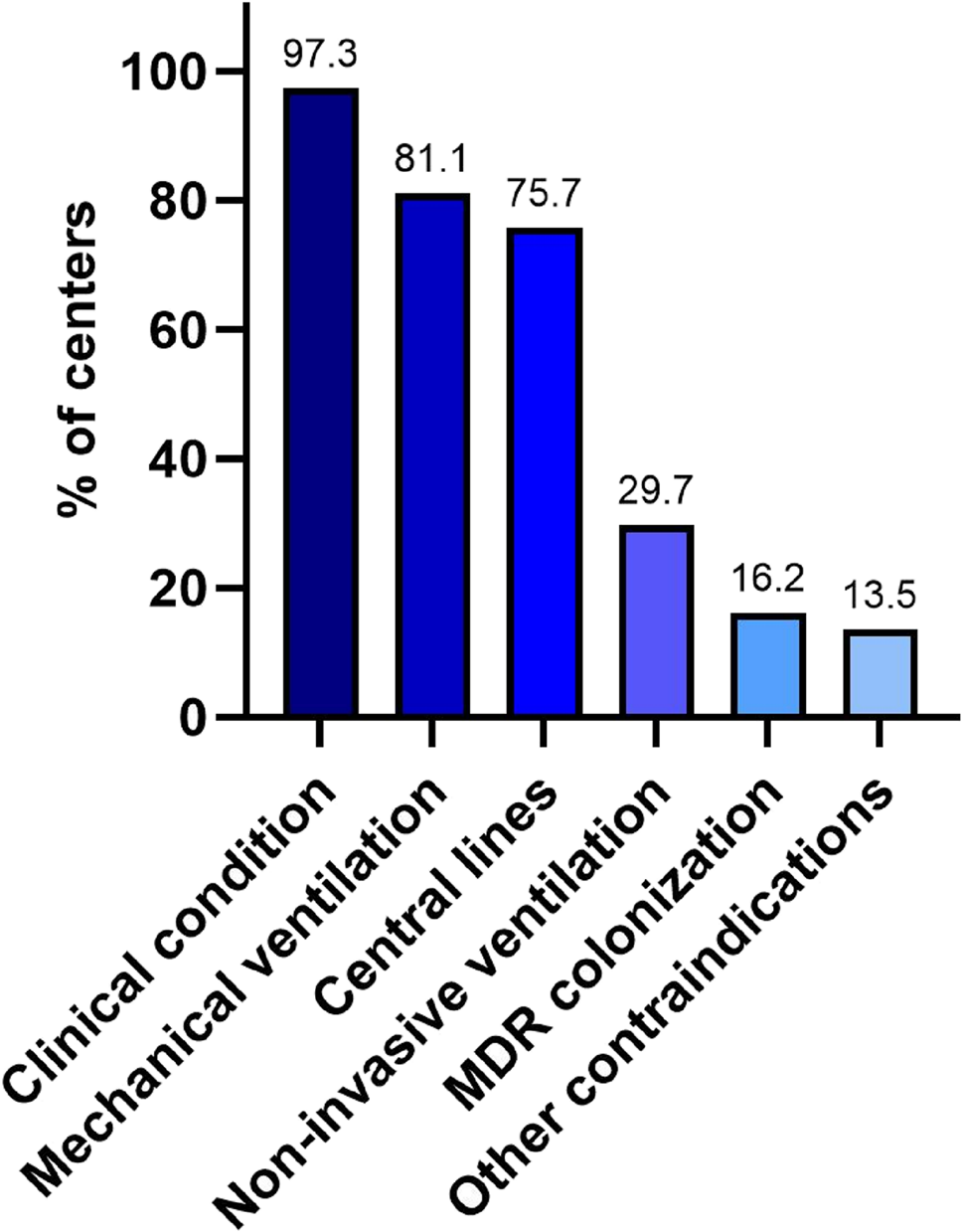
Contraindications to bathing as reported by the centers practicing bathing (*n* = 37; multiple answers allowed)

The criteria used to determine whether an infant was bathed included clinical condition (81.1%), postnatal age (56.8%), gestational age (48.6%), birth weight (16.2%), and other factors (8.1%), with multiple responses allowed. Common contraindications are presented in Fig. 2.

Clinical condition, referring to clinical instability, was the most reported contraindication (*n* = 36 centers; 97.3%), followed by mechanical ventilation (*n* = 30; 81.1%), and presence of central lines (*n* = 28; 75.7%).

When asked about the use of bathing additives, only 31 of the 37 centers practicing bathing provided responses. Sixteen of these centers (51.6%) reported not using any additives routinely. Among those that did, almond oil was most frequently used (*n* = 6; 19.4%), while some centers reported using mother´s milk, olive oil, Hipp^®^ bathing additive, or other oils.

Bathing was more frequently reported in smaller centers (85% vs. 68%), which also reported central lines less often as a contraindication (64% vs. 93%). In other European centers, bathing was practiced in the majority (*n* = 7; 87.5%) with varying frequencies.

### Skin antisepsis and disinfection

Responses regarding skin antisepsis were received from only 43 centers (90%). Octenidine 0.1% was the most commonly used disinfectant (*n* = 33; 76.7%), followed by a combination of octenidine 0.1% with propanol and isopropanol (alcoholic solution, Octeniderm^®^; *n* = 9; 20.9%) and a combination of octenidine 0.1% with phenoxyethanol (non-alcoholic solution, Octenisept^®^; *n* = 6; 14.0%). Multiple answers were permitted, and some centers reported differentiated use depending on gestational age, postnatal age, or birth weight. One center reported using propanol alone. Chlorhexidine was not reported by any center.

Skin disinfection practices in other European centers also varied: chlorhexidine (either aqueous or alcoholic) being reported by five centers (62.5%), along with other agents like Prontosan^®^ (polyhexanide, undecylenamidopropyl-betaine), Kodan^®^ (propanol), and Octenisept^®^ (octenidine 0.1%, phenoxyethanol).

### Main challenges in skin care

**Fig. 3:**
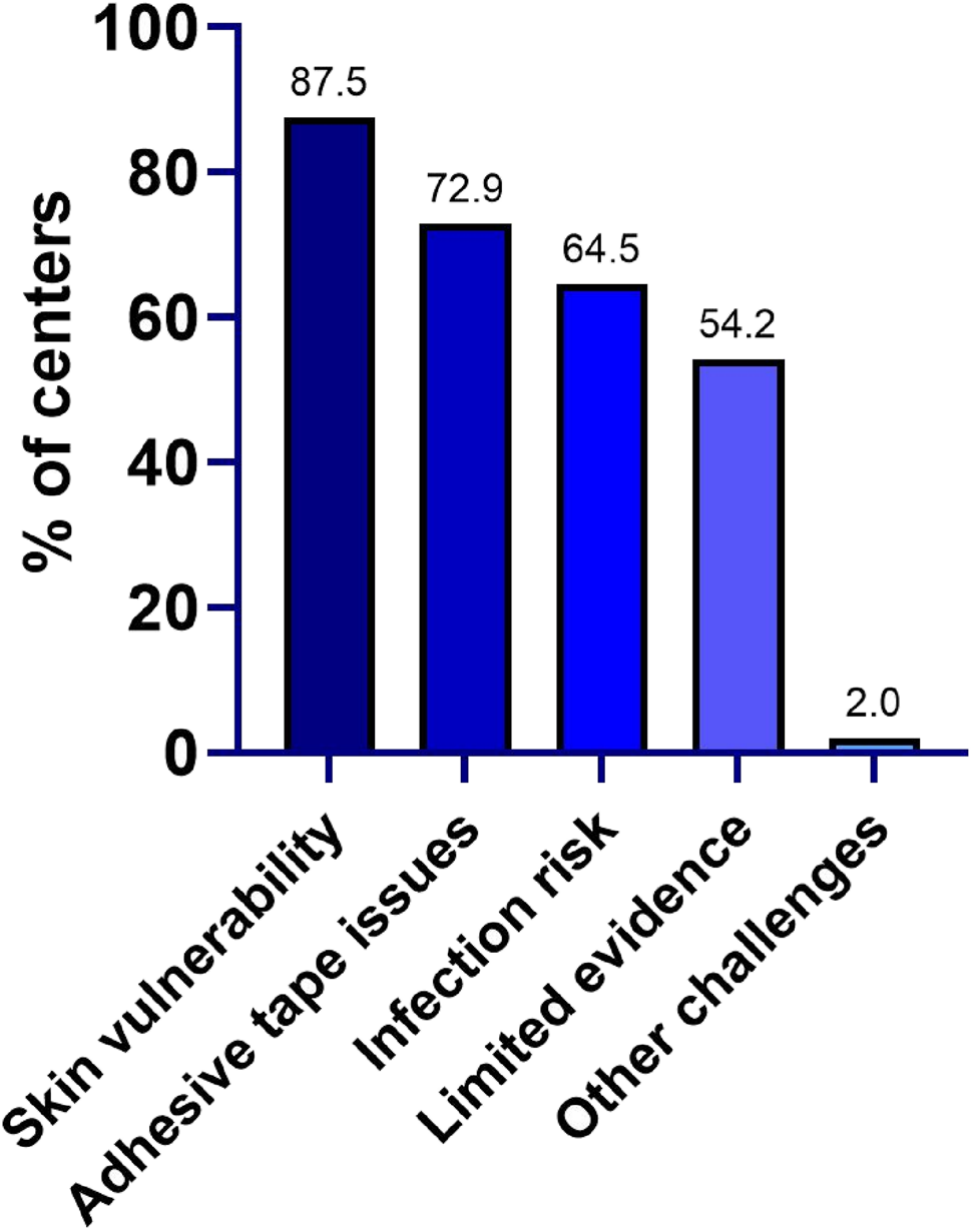
Challenges in skin care in VLBWI. Multiple answers were permitted

Figure 3 illustrates the main challenges in skin care for VLBWI as reported by German NICUs. High skin vulnerability was identified the most significant challenge, reported by 87.5% (*n* = 42) of centers.

When asked about criteria guiding the selection of skin care products in NICUs, centers reported that clinical evidence and practical experience were important factors (*n* = 29; 60.4% each). Hygiene regulations were reported by 54.2% (*n* = 26) of centers, followed by other criteria (*n* = 4; 8.3%), and parental preference (*n* = 3; 6.3%).

In other European centers, the main challenges perceived in skin care were similar to those reported in Germany, with all centers (*n* = 8; 100%) identifying high skin vulnerability, and *n* = 7 (87.5%) noting the risk of infection, and issues with adhesive tapes, respectively. However, only one center (12.5%) reported limited or unclear evidence as a challenge. Criteria guiding the selection of skin care products included clinical evidence (*n* = 6; 75%), practical experience (*n* = 5; 62.5%) and hygiene regulations (*n* = 2; 25%).

## Discussion

This study provides an up-to-date overview of current skin care practices among German NICUs and highlights substantial variability across centers. Although an international survey has previously explored global practices, contemporary national data from Germany have been lacking. Our study closes part of this gap by also presenting data on bathing practices and the challenges neonatologists encounter when caring for the skin of preterm infants and selecting appropriate skin care products.

Only a small number of centers in our survey reported using a standardized skin risk assessment tool or a standardized skin care protocol (14.6% and 17%, respectively). In contrast, a worldwide survey found that 72% of centers had local skincare guidelines for extremely preterm infants [5]. In a survey among German NICU nurses from 2022, 73.4% of nurses reported having local skin care regulations or protocols [15]. The lower proportions in our study may partly be due to our specific focus on “standardized” tools or protocols, which was not further defined, or differences in how the question was interpreted by clinicians. Because of the lack of national guidelines for preterm skin care or skin risk assessment, some units may rely on internal practices that are not formally reported as “standardized” and therefore were not captured here.

A national German expert standard on skin care explicitly excludes preterm infants from their recommendations [10]. Our findings therefore underscore both the significant variability in practice and urgent need for further research to inform standardized national or international recommendations. Globally, any neonatal skin risk assessment tool, the Neonatal Skin Condition Score and the Braden Q scale were reported as the most frequently used assessment tools (22%, 17% and 14%, respectively), with only 18% of European countries reporting no use of any tool. Regular skin assessment every four hours was associated with a reduced risk of skin injury [5]. In contrast, most German centers in our study reported no structured assessment tool, and among the few that did, most applied them only once daily. Again, it is likely, however, that frequent informal nurse assessment occurs during routine care of VLBWI; our survey did not capture these non-standardized evaluations.

Another major reason for the limited use of structured tools may be that most tools have not been validated for VLBWI [6]. Although tools such as the Neonatal Skin Risk Assessment Scale have shown promising predictive values, they remain under evaluation and require further validation in this specific population [16]. Collectively, the lack of validated tools emphasizes the need for systemic development and validation of skin risk assessment instruments tailored for VLBWI balancing reliable detection of risk with minimal handling. A standardized skin risk assessment could subsequently guide skincare practices.

Regarding skin care products, we observed substantial variability among German NICUs. In our study, 60.4% of centers reported using at least one skin care product with considerable heterogeneity in the specific products chosen. Similar variability has been reported in survey from other regions [5, 17]. Globally, 41% of centers reported using emollients, with substantial geographic and income-related differences. Emollient use was highest in extremely preterm infants in African countries (63%), whereas global use in high-income countries was only 27% [5]. European centers reported emollient use in 45%, which is more consistent with our findings. Differences in populations in both studies must be considered, however, as the global survey focused on preterm infants < 28 weeks gestational age, whereas our study focused on VLBWI (< 1500 g birth weight) [5].

Globally, oil-based emollients are more commonly applied than petrolatum-based products [5]. Evidence suggests, that oil-based emollients may reduce infection risk [7, 18] and support weight gain [19, 20], although these benefits largely apply to low- or middle-income settings [7, 19]. A multicenter trial in Australia and New Zealand (COSI-2 trial) is currently examining whether coconut oil reduces infection in extremely preterm infants [8]. Other positive effects have been seen in several studies in reduction of skin irritations, heat and water loss and improved stratum corneum hydration [2, 21]. However, also negative effects such as skin irritation, disruption of skin barrier or contamination have been reported [21]. Petrolatum-based emollients though have partly been associated with higher risks, including infections (e.g., coagulase-negative staphylococci, Candida), tissue injury, interference with adhesives, and environmental contamination [5, 18]. The WHO guideline for care of preterm of low-birth-weight infants states, that “application of topical oil to the body of preterm of low-birth-weight infants may be considered (Conditional recommendation, low-certainty evidence)”. Moderate benefits were seen regarding decreased severe infection (low-certainty evidence), increased weight (low-certainty evidence), and increased length (moderate-certainty evidence) when comparing use of topical oil vs. no topical oil [22].

In our survey, Bepanthen^®^ emerged as the most frequently used skincare product in German NICUs. It is an oil-in-water emulsion containing dexpanthenol and lanolin. However, there are only limited data in the literature regarding its use in preterm infants. One study reported beneficial effects in the prevention of dermatitis, although it was found to be less effective than olive oil alone [23].

The use of natural oil-based emollients varied across centers, with sunflower oil being the most commonly reported. Lanolin, an occlusive emollient, was used as a standalone product in only 10% of centers; however, it is also included in combination products such as Bepanthen^®^. Despite ongoing large-scale studies investigating coconut oil in Australia and New Zealand, none of the German centers reported its use.

More than half of the responding centers identified limited or unclear evidence of skin care products as a major challenge in preterm skin care, further emphasizing the need for high-quality studies on emollient use in the European setting, especially in VLBWI.

Bathing is now common practice in German NICUs, although bathing frequency and the additives used varied widely. A German survey from 2021 reported that 88% of level I NICUs performed bathing [15], compared with 77% of centers in our survey. This difference may reflect our explicit focus on VLBWI rather than all neonates, or center-level bias. Current recommendations suggest bathing every four days [18, 24–26]. Tub bathing is less likely to cause temperature instability than sponge bathing [18, 23]. However, hygienic measures need to be taken in account, as most NICUs have shared rooms for multiple infants. Interestingly, NICUs that did not practice bathing, reported the highest level of hygiene concerns [15]. Swaddled bathing appears to reduce stress and temperature loss in preterm infants [27]. Overall, however, high-quality evidence on bathing practices in high-income settings remains very limited [15, 18].

For skin antisepsis, most centers used octenidine 0.1%, consistent with national guidelines recommending its use in VLBWI during the first two weeks of life [28, 29]. Some centers reported using octenidine in combination with propanol and isopropanol, or phenoxyethanol (e.g., Octeniderm^®^, Octenisept^®^), depending on gestational and postnatal age. Our data align with a recent German survey showing predominant use of octenidine 0.1% with some units using octenidine plus phenoxyethanol [28], when implementing central venous catheters in the first weeks of life. Alcoholic solutions were rarely used, likely reflecting the early postnatal age and the vulnerability of extremely preterm infants receiving catheters. In our survey, the alcoholic solution (Octeniderm^®^) was reported by roughly 21% of centers, with some centers giving detailed data on specific gestational or postnatal ages, likely not using it routinely within the first two weeks of life.

Compared with a 2012 German survey, the use of octenidine 0.1% without phenoxyethanol seems to have increased (76.7%; previously 58%) with decreased use of Octenisept^®^ and Octeniderm^®^, and no reported use of povidone-iodine and chlorhexidine [30]. Although the populations studied differ (VLBWI vs. extremely preterm infants), it is notable that adverse skin reactions were still reported in 27% of extremely preterm infants despite octenidine 0.1% use [30]. In contrast, international and European practice shows broader use of alcoholic or non-alcoholic chlorhexidine in our survey and in other recent reports [5]. Worldwide, chlorhexidine (aqueous or alcoholic), iodine-based solutions, and alcohol–antiseptic combinations remain the most frequently used agents [5]. These agents are not recommended to use in German NICUs anymore due to concerns about transdermal absorption [28, 30] and were not reported by any German center.

Across all aspects of skin care, the most commonly cited challenges were the high skin vulnerability of VLBWI, issues related to adhesives, and infection risk. When selecting skin care products, both clinical evidence and practical experience played equally important roles (each cited by 60.4% of centers), and local hygiene regulations were emphasized by more than half of centers (54.2%).

Interestingly, when compared with a U.S. survey from 1999 [31], the heterogeneity among centers or countries regarding skin care practices in preterm infants has not significantly decreased [5]. While in other regions of the world, such as Switzerland and Western Australia, official skin care guidelines have been implemented, some of which aim to harmonize skin care practices across selected national centers or include recommendations on skin risk assessment, bathing and ointments [11–13], this is not the case in Germany.

Thus, further research is needed to develop validated skin risk assessment tools and optimized, standardized, yet individualized, skin care approaches for this vulnerable and heterogeneous population. Considering the many confounding factors involved (e.g., incubator humidity, skin care products, skin-to-skin care, invasive procedures), this remains a major but necessary challenge in addressing the currently neglected field of premature skin.

To our knowledge, this is the only report describing current skin care practices for VLBWI in Germany. Highlighting the considerable variability and lack of standardization at the national level is essential to improving care for this vulnerable population.

A limitation of this study is the potential restriction in representativeness, related to voluntary participation, only partial coverage of NICUs caring for VLBWI in Germany, and the limited availability of detailed institutional characteristics. We estimated 9,000 to 10,000 VLBWI born annually in Germany [14] and estimated a coverage of approximately 25% of these VLBWI by the participating centers. However, estimates of total numbers were derived from categorical data using midpoint approximations and should therefore be interpreted as indicative rather than exact values. A potential source of bias may be that not all Level III NICUs were contacted; currently, there are approximately 160–170 in Germany, and the responding centers therefore represent around 30% of all Level III NICUs. For some aspects, such as ‘skin vulnerability’ or ‘standardized protocol’, no clear definitions exist or were provided in our survey; therefore, response bias may have arisen due to different interpretations of these questions.

Although responses were checked for duplication based on country, annual VLBWI numbers, and detailed survey content, duplicate participation cannot be completely ruled out. Moreover, our limited number of European responses is not representative of the region but provides additional insight, suggesting that variability is likely not unique to Germany and aligns with previous reports demonstrating similar heterogeneity [5]. From our data, we cannot determine whether the observed heterogeneity in skin care has adverse effects on the skin integrity of VLBWI. Also, we did not stratify for different gestational age categories within the group of VLWBI or different bathing methods. Further surveys and research should therefore focus on these aspects and include stratification of subgroups within this highly vulnerable population of VLBWI. Although we asked about perceived challenges in VLBWI skin care and product selection, our data do not allow us to determine, whether the reported heterogeneity among centers primarily reflects differences in awareness and/or local implementation of skin care standards, or rather uncertainty due to lack of evidence.

## Conclusion

Skin care practices for VLBWI in German NICUs vary widely. Our survey results may contribute to increased awareness of this issue. However, from our data, we cannot determine whether the observed heterogeneity in skin care has adverse effects on the skin integrity of VLBWI. High skin vulnerability, issues with adhesives and infection risk remain the main challenges reported. There is the urgent need for stronger evidence, ideally from randomized controlled trials, on optimal skin development, skin risk assessment and optimized skin care. Such data are essential to standardized yet individualized approaches for this highly vulnerable population.

## Supplementary Information

Supplementary Material 1.

## Data Availability

All data are available from the corresponding author upon request.

## Abbreviations

NICU: Neonatal Intensive Care Unit
VLBWI: Very low birth weight infants
