# Supplementary material for: Current skin care practices in very low birth weight infants in German Neonatal Intensive Care Units – results from a cross-sectional survey
Source: Mol Cell Pediatr. 2026 Apr 29;13:23. doi: 10.1186/s40348-026-00237-0 (PMC13129118; doi:10.1186/s40348-026-00237-0)
Supplement: Supplementary file 1 — Supplementary Material 1. [file 40348_2026_237_MOESM1_ESM.pdf]

Bitte so markieren: ☐ ☒ ☐ ☐ ☐ Bitte verwenden Sie einen Kugelschreiber oder nicht zu starken Filzstift. Dieser Fragebogen wird maschinell erfasst.
Korrektur: ☐ ■ ☐ ☒ ☐ Bitte beachten Sie im Interesse einer optimalen Datenerfassung die links gegebenen Hinweise beim Ausfüllen.

1. NICU facts

1.1 Which country are you from?

1.2 How many very low birth weight infants (<1500 g birth weight) do you treat annually?
☐ < 25 ☐ 25 - 50 ☐ 51 - 75
☐ 76 - 100 ☐ > 100

2. Skin care

2.1 Are any skin risk assessment scores used in your unit?
☐ Yes ☐ No

2.2 Which scores?

2.3 How often are they applied?
☐ daily ☐ several times a week ☐ weekly
☐ if necessary

2.4 Does your institution have a standardized skin care protocol for preterm infants?
☐ Yes ☐ No

2.5 Please describe shortly

2.6 Are skin care products routinely used?
☐ Sunflower oil ☐ Other oil ☐ Bepanthen (dexpanthenol)
☐ Lanolin ☐ Other products ☐ No routine application

2.7 Which other oil or product?

2.8 Which products are used for skin disinfection?

3. Bathing practices

3.1 Do you practice bathing in your NICU?
☐ Yes ☐ No

3. Bathing practices [Fortsetzung]

3.2 Which criteria are decisive for the bathing?
- ☐ Gestational age
- ☐ Birth weight
- ☐ Postnatal age
- ☐ Clinical condition
- ☐ Other

3.3 How frequently are very low birth weight infants bathed?

3.4 Which bathing additives are used?

3.5 Are there any contraindications to bathing?
- ☐ Clinical condition
- ☐ Central lines
- ☐ Mechanical ventilation
- ☐ Non invasive respiratory support
- ☐ Microbial colonization with multiresistant strains
- ☐ Other

4. Challenges in skin care

4.1 What are the main challenges in skin care of very low birth weight infants?
- ☐ High skin vulnerability
- ☐ Issues with adhesive tapes or fixation
- ☐ Risk of infection
- ☐ Limited or unclear evidence
- ☐ Other

4.2 Please specify

4.3 Which criteria are most relevant for product selection in your unit?
- ☐ Clinical evidence
- ☐ Practical experience
- ☐ Parental preference
- ☐ Hygiene regulations
- ☐ Other

4.4 Please specify

4.5 Any other comments or information from your side?
